# ‘I want to be generous, but I only have limited energy’: a qualitative study of amyotrophic lateral sclerosis patients’ preferences for clinical trials participation

**DOI:** 10.1080/07853890.2025.2586150

**Published:** 2025-11-11

**Authors:** Morolake J. Adeagbo, Justin Kahler, DeShauna Jones, Heather Schacht Reisinger, Andrea Swenson

**Affiliations:** ^a^ Institute for Clinical and Translational Science, University of Iowa, Iowa City, Iowa, USA; ^b^ Health and Human Physiology, University of Iowa, Iowa City, Iowa, USA; ^c^ Iowa City VA Health Care System, Iowa City, Iowa, USA; ^d^ Carver College of Medicine, Department of Internal Medicine, University of Iowa, Iowa City, Iowa, USA; ^e^ Carver College of Medicine, Department of Neurology, University of Iowa, Iowa City, Iowa, USA

**Keywords:** Amyotrophic lateral sclerosis (ALS), emotions, clinical trials, qualitative research

## Abstract

**Background and objective:**

Research and decisions on health-related issues such as Amyotrophic Lateral Sclerosis (ALS) continue to evolve as the etiology of network degenerative disease remains indeterminate. Due to its heterogeneity, clinical trials (CTs) are continually being conducted for beneficial breakthroughs aimed at improving the lives of patients with ALS. However, there is a dearth of research on ALS patients’ health-seeking decisions and preferences in CTs, particularly for patients living in rural areas. To bridge this important gap, we explored patients’ subjective experiences and preferences in CT participation through their emotions.

**Materials and methods:**

Seventeen participants (10 ALS patients, 6 healthcare professionals, and an advocacy group representative associated with ALS) affiliated with the University of Iowa ALS Multidisciplinary clinic were purposively selected and interviewed for the study. Qualitative descriptive data were analyzed using thematic content analysis to understand the patients’ experiences and preferences.

**Results:**

Findings indicate key emotional and logistical challenges including fatigue, travel distance and constraints, limited trial availability, which are exacerbated by the disease’s rapid progression and restrictive eligibility criteria. Participants’ narratives highlighted frustration, anxiety, and fear as central emotional experiences influencing their health-seeking decisions. Conversely, expressions of hope and empathy emerged as significant motivators, with patients demonstrating a willingness to participate in CTs despite the known risk of limited personal benefits, while focusing on the need to benefit future ALS research. Patients prefer and desire more compensation, broader eligibility and inclusive criteria, trial availability and publicity, and access to telemedicine.

**Conclusion:**

Given the multifaceted physical, and emotional challenges faced by ALS patients, this study recommends prioritizing patient preferences in future CTs and intervention designs, while advocating for targeted grants and sustained funding that supports ALS clinical trials. This will better align with the needs and expectations of ALS patients, thereby enhancing trial participation and overall patient satisfaction.

## 1. Introduction

Clinical trials remain one of the most important pathways for testing the safety and efficacy of medical interventions in patients. Guided by the International Conference on Harmonization (ICH) principles on product safety, efficacy, and quality, clinical trials are aimed at improving and informing novel approaches to screening and diagnosis, prevention, and treatment of different diseases, such as Amyotrophic Lateral Sclerosis (ALS) [1,2]. ALS is a neurodegenerative disease that progressively affects spinal, cortical, and brainstem motor neurons, causing muscle weakness, muscle atrophy, and ultimately respiratory failure. Most patients die within 2–4 years after diagnosis [3–5]. In the United States, approximately 3 to 5 in 100,000 people are currently diagnosed with ALS, while 1 to 2 persons per 100,000 are diagnosed each year [6–8].

As the etiology of ALS remains indeterminate and due to the heterogeneity of this disease, clinical trials are continuously conducted for beneficial breakthroughs aimed at improving ALS patients’ lives [9]. For instance, some ALS-related clinical trials and therapeutic options such as Riluzole and Edaravone target the suppression of motor neuron degeneration and loss, while transcranial direct current stimulation (tDCS) and repetitive transcranial magnetic stimulation therapy (rTMS) also aim to potentially reduce motor neuron progression and regulate cortical excitability [10–12]. However, the pace and number of therapeutic breakthroughs remains limited due to a combination of interpersonal, intrapersonal, operational and systemic factors [13,14]

Participation among individuals living with ALS remains low and varies considerably across trial sites, particularly in the United State where the estimated enrollment rate is approximately 10% [13,15]. People living with ALS often face significant barriers to clinical trial enrollment, including limited access due to geographic distance to trial sites, restrictive eligibility criteria, fear and uncertainty about disease progression, financial and logistical burdens, skepticism about the potential benefits of experimental treatments compared to current standards of care, and sometimes participation fatigue [13,14,16]. From a broader perspective, systemic challenges further limit the effectiveness and reach of ALS clinical trials. These include the rapid and variable progression of the disease, significant heterogeneity in how ALS presents across individuals, limited funding and infrastructure to support trials [7,13]. Additionally, the underrepresentation of minorities, and individuals from rural populations also limits access to ALS clinical trials and outcomes [7,13].

While ongoing efforts aim to expand clinical trials availability despite the challenges, a cure or consistently effective treatment for sporadic ALS remains unknown [17–19]. This underscores the significant need for inclusive, well supported clinical trials to find treatments that can significantly slow disease progression and ultimately cure ALS. As the findings from past trials and research continue to lay the foundation for ongoing trials that aim to improve survival or discover a curative breakthrough in ALS research [6,20], there remains limited literature on the preference and influence of ALS patients’ decisions regarding enrollment and participation in clinical trials, especially within rural settings. This gap highlights the importance of understanding patients perspectives, preferences and influences to ensure that future clinical trials are accessible and responsive to the important needs of the ALS community.

To address the limited understanding of ALS patients’ health-seeking decisions, the objective of this study was to qualitatively explore patients’ experiences and how these influence their clinical trial preferences, motivations and willingness to participate. Guided by the interpretivist lens, the study focused on the subjective responses to uncover how emotional indications, that are interpreted through patients’ narratives inform their preferences, motivations, and willingness to participate in clinical trials [21]. This approach recognizes that health decisions and outcomes are shaped by causal links between different factors, such as social and economic, environmental, behavioral, and judgment, which are often guided by emotions in diverse contexts [22–25]. Understanding these influences is essential for identifying barriers and health-seeking decisions which will help facilitate and promote more patient-centered strategies for ALS clinical trial engagement.

Positioning our argument within the ‘causes of causes’ context, we argue that emotions are key determinants of health and health decisions while considering other related influences [22–25]. As the healthcare system and its human actors are guided by a relational structure, it is critical to consider the influence of emotions in this setting. For instance, patients’ emotions, emotion regulation strategies, role and trust in medical practitioners have been identified as primary factors that influence cancer patients’ health-seeking behavior and decision-making in clinical trial participation [26–29]. Similarly, understanding the emotional drivers behind ALS patients’ responses can offer valuable insights into their health-seeking behaviors. While emotions alone may not resolve enrollment challenges, recognizing their influence is critical to developing more inclusive and patient-centered approaches to ALS clinical trial design and engagement.

## 2. Materials and methods

This study utilized qualitative methods to explore and elicit in-depth perspectives on ALS patient preferences and participation in clinical trials [30,31]. Participants included patients diagnosed with ALS and receiving care at the University of Iowa ALS Multidisciplinary Clinic. The clinic serves a large catchment area with patients traveling an average of 180 miles for one in person clinic visit. Other participants were key stakeholders such as genetic counselors, therapists, neurological research nurse coordinators, and ALS advocacy group representatives.

This study was approved by the University of Iowa Internal Review Board [IRB#202001086]. Notably, no formal hypothesis was proposed due to the study’s qualitative and exploratory design. In contrast, a well-defined objective guided the development of the data collection tool and thematic analysis. The interview guide, which consisted of open-ended questions, was developed through a comprehensive process of clearly defined objectives and a thorough review of literature to identify key gaps in ALS patients’ clinical trial participation, pilot tests and expert review. The finalized guide focused on patients’ experiences, opinions, and attitudes towards clinical trials, challenges to patient enrollment and facilitators of participation in clinical trials, and the overall context in which patients and stakeholders make decisions regarding clinical trials. Some of the questions include:

*ALS Patients*: What have you considered in regard to research participation? What factors contributed to your decision [not] to participate in the study? What are the benefits you see in patients participating in ALS clinical trials? What, in your opinion, can be done in developing trials to make them more appealing to patients?

*ALS Key stakeholders*: What are some of the barriers that exist for ALS patients? Describe previous experience with patients enrolled in ALS clinical trials. What barriers do ALS patients face in participating in clinical trials? What, in your opinion, can be done in developing trials to make them more appealing to patients? What makes it difficult for you to enroll ALS patients in clinical trials? (Factors related to the study? Clinical factors? Individual patient factors)

Given the homogeneity and vulnerable hard-to-access context of the sample, individuals with sufficient and high information power were purposively approached and recruited for the study between November 2020 and June 2022 [32–34]. The specificity of the samples and depth of information possessed by the sample were considered based on the health and work-related experiences of the patients and stakeholders, respectively [34]. Recruitment was inevitably constrained by the COVID-19 restrictions. With the approval of the consultant neurologist and recommendation of the ALS multidisciplinary clinic medical director, official IRB approved letters/emails, and the study information sheet was sent to potential participants who utilized the University of Iowa ALS Multidisciplinary Clinic. The staff in the ALS clinic, ALS study team members, and ALS local patient advocacy group leadership also received email explaining the study. Individuals signified interest by responding to the emails. Then, to ensure participants’ adequate comprehension of the study, a member of the qualitative research team (JK) also discussed the study, and the element of consent with identified and interested participants *via* phone. Participants were informed that responding to the email and agreeing would be considered as informed consent to interview and possible publication of the collected data.

Prior to the commencement of the interview, the interviewer explicitly asked each participant if they consented to be interviewed, and audio recorded. After obtaining consent from each participant, the interviews were conducted by trained qualitative researchers (JK and DJ), audio-recorded for verbatim representation, and anonymously transcribed. Verbal consent was obtained to confirm the participants’ willingness to participate in the study. The semi-structured in-depth interview sessions ranged between 30 and 60 min and were conducted both physically in the clinic and virtually *via* the Zoom^TM^ communication application. The participants were asked to participate in only one session. Due to cognitive, physical, or communication impairment (dysarthria) due to disease progression, family members or caregivers were permitted to attend, provide support, help with recollection, and validate patients’ answers to questions specific to patients if they were unable to answer fully. However, caregivers were not the primary participants. Additionally, a typed response to the interview question was used to accommodate one patient’s condition.

Demographic information and anonymized transcripts of the interviews were reviewed (JK, DJ, HSR, MJA) for accuracy and uploaded to MAXQDA, a qualitative data management and analysis software. Preliminary codes were first generated (JK, DJ, and HSR) using inductive and deductive qualitative analysis approaches [35,36]. Using the same approach, while incorporating the interpretivist approach guided by the important components of emotion, a second round of coding was conducted (MJA) to establish and confirm related codes and categories of themes [21]. The process entails the analysis of all participants’ narratives as textual data, while focusing on the verbal patterns that reveal underlying emotional experiences and expressions [29]. After completion, two analysts (HSR and MJA) discussed and resolved discrepancies. The process enhanced rigor and ensured research validity, as the overarching themes and subthemes were verified against quotes to accurately represent participants’ responses. Subsequently, data analysis generated themes that reflected barriers, facilitators, preferences, and recommendations. The final codebook presents each quote and subtheme to show the depth of the themes.

## 3. Results

Ten patients with ALS, six healthcare workers, and a representative from a patient advocacy group were interviewed. The demographic details of the interviewed patients and stakeholders are presented in (Tables 1 and 2) respectively.

**Table 1.** Participants demographics (patients).

Variables	Components	Frequency (%)
Gender	Men	5 (50%)
	Women	5 (50%)
Clinical trial participation	Yes	4 (40%)
	No	6 (60%)
Travel distance (hospital visit)	30 minutes – ≥ 120 minutes	Each in person visit

**Table 2.** Participants demographics (stakeholders).

Work experience (ALS-related)	*n*
<5 years	1
5–10 years	2
10–20 years	2
20–30years	2

**Figure 1.**
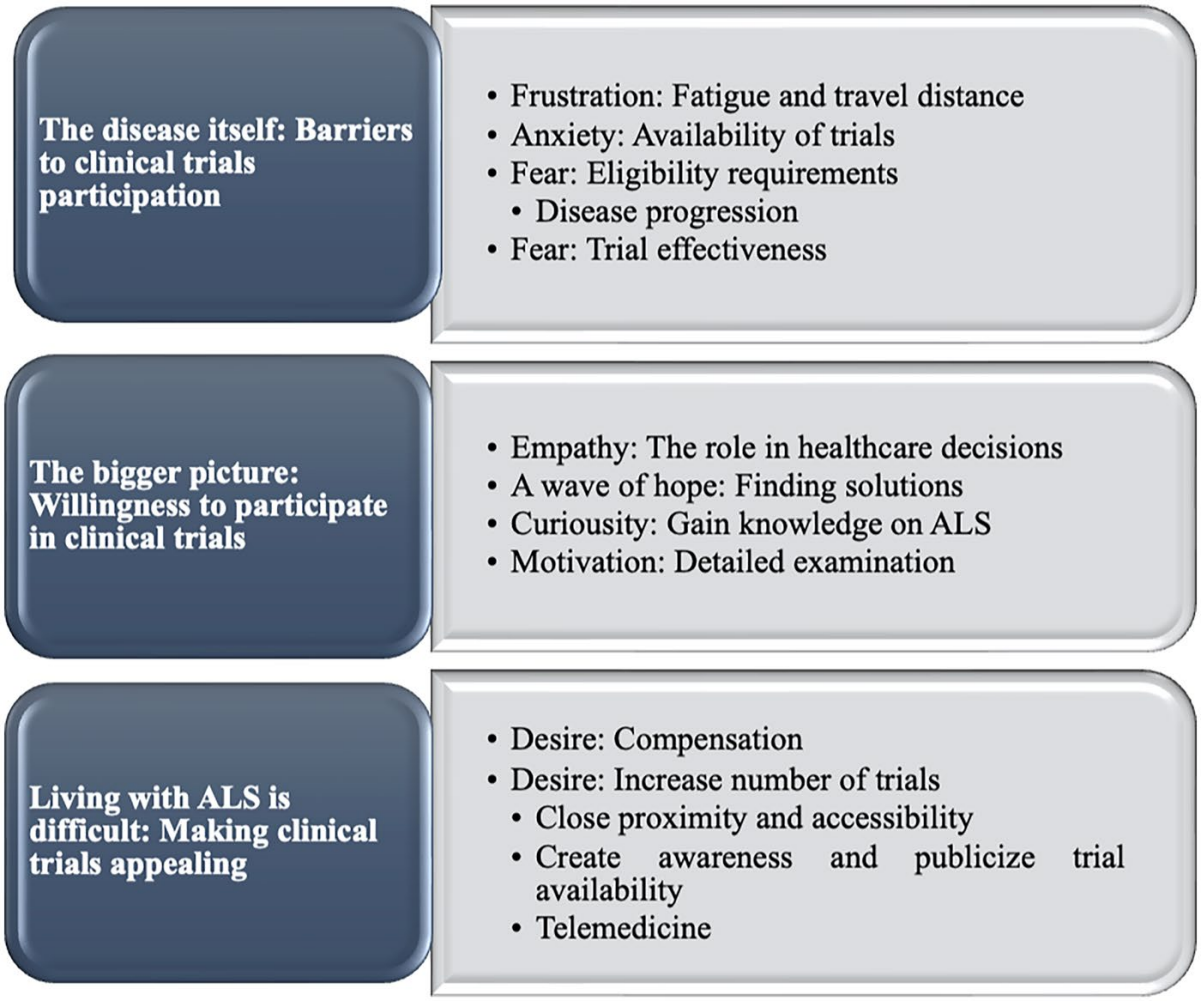
Framework of thematic analysis (see the supplementary material for more detailed thematic analysis). This figure illustrates the structured approach used in the thematic analysis of the study. It demonstrates how the subthemes align to inform the identification and analysis of each overarching theme. Each theme and the correlating subthemes are presented in separate boxes across each other to provide a comprehensive overview of the analysis.

To adequately understand the preferences and health-seeking decisions regarding ALS patient participation in clinical trials, this study explored the role of patients’ emotions. The patients’ narratives were corroborated by key stakeholders’ perspectives to validate and strengthen the findings of the study. Following the analysis, three pertinent themes are presented in Figure 1 and discussed further below.

### 3.1. The disease itself: barriers to clinical trial participation

Daily life is difficult for individuals with ALS, and this unavoidably affects their enrollment in clinical trials. The impact of the disease on physical functioning is a major hurdle for patients, expressed through frustration, fear, and anxiety. Fatigue is a general narrative of patients. Most of the patients shared their willingness to participate in ALS clinical trials but were mostly restricted by the resulting fatigue and limited energy. A patient who did not participate in the clinical trials shared:

‘I want to be generous, but I only have limited energy.’ (P9_NP).

The physical impact caused by fatigue, as the disease progresses, also contributes to travel difficulties. As the University of Iowa is committed to caring for rural populations from within Iowa and its surrounding states, most of these patients have to travel long distances for each in person clinic visit. As a result, most patients are presented with the frustration of traveling long distances, combined with the physical impact of the disease.

For most patients, trial availability is another barrier that causes frustration and anxiety. Most patients wished that more trials have broader eligibility and inclusive enrollment, especially as ALS patients are often restricted due to variability in progression. This means that those who are aware of the available trials and are willing to participate are either told to wait or are often not eligible for various phases of the few available clinical trials. Waiting and accessibility often increases anxiety and fear. From another perspective, waiting to be selected can also be frustrating; however, not obtaining the desired result after enrolling in a clinical trial can be more disappointing. Even though patients who had been involved in clinical trials confirmed that they were adequately informed and prepared for the possibility of not getting the desired outcome, most shared their concerns and frustration. These expectations often lead to frustration, disappointment, and reluctance, which influence their decisions and interests in participating in future clinical trials. This issue is perceived as a barrier, as it results in reluctance, which may ultimately impact patients’ participation in clinical trials. One patient who had participated in a previous clinical trial shared the following:

‘It was just kind of disheartening knowing it didn’t do anything.’ (P7_PCT)

These findings on patients’ struggles and their need for relief/cure emphasize the often-unmet expectations of ALS patients. With the validation of the stakeholders, these major barriers were summed up by one stakeholder as:

‘Unfortunately for this group there are several barriers. They are very anxious about participating in the trials. However, many of our patients do not live locally. Most of them do not, so transportation to and from hospitals can be difficult. As patients progress with ALS, they become fatigued more easily, so visits for research trials can be lengthy […] Some patients right off the bat want to be included in research trials as soon as they are diagnosed. However, more often than not, patients just need to wrap their heads around it and get their lives in order. Once they start progressing, they think about the research trials. The difficulty with this is that many trials have these criteria that the patients have to meet. The major one is breathing. They must be above a certain percentage in this breathing assessment. This has been a challenge because patients sometimes progress rapidly, and by the time they are coming into a research trial, they may not qualify.’ (ALS Stakeholder_7)

However, despite the barriers to clinical trial participation presented through patients’ narratives of anxiety, fear, and frustration, most patients also shared their willingness to participate in clinical trials in the near future.

### 3.2. The bigger picture: willingness to participate in clinical trials

This theme demonstrates the participants’ ability and readiness to choose despite the complexity of living with ALS. Most patients expressed their willingness to participate in clinical trials. This theme developed from the subthemes that highlight the important roles of empathy and hope in health-related decisions, participants’ curiosity, and being motivated by detailed examinations that could lead to positive health outcomes. Presented in their narratives, the patients were well informed about the need to find a cure for ALS and were aware of some of the inconclusive outcomes of prior clinical trials. Despite this knowledge, the analysis highlights two strong and connected emotions, hope and empathy. Hope was emphasized as a driving force for participation, whether in the past or in future clinical trials. This is because of the value they place on finding relief or cure, and how their expectations may be met through clinical trials. According to a patient:

‘In hopes that maybe there was something that would help slow the progression and if not, at least help gather information that maybe down the road that would help, if not me, somebody else. I mean, we knew it was not going to be a cure, so it was just to help somebody else. That’s what we talked about […] yeah, it was not the silver bullet, so to speak, but we knew maybe there was a chance.’ (P7_PCT)

The representation of hope emphasizes patients’ need to find relief from ALS, not only for themselves, but also for helping others. Their willingness to contribute to ALS discovery and innovation through participation in clinical trials highlights the role of affective and cognitive empathy in health spaces, as it shows the comprehension of the needs of others and their willingness to be generous through participation in trials. A patient stated that:

‘My main consideration was, even if it did not help me, it might help someone in the future, so that was always on my mind… I wanted to be helpful and hopefully get help myself.’ (P4_PCT)

From a related but distinct perspective, another patient perceived participation in a clinical trial as an opportunity to rule out unsuccessful tests, which may enable better prospects of a breakthrough in ALS research:

‘Well, I know that it’s probably not likely that it’s necessarily going to help me specifically, but even if this whatever I try is something that doesn’t work, it’s just one more thing that we know doesn’t work. Trying to get that much closer to answers, doing whatever I can to help with that […] It’s one of those– like I want to participate because I want to be helpful in whatever way that I can. And if it helps me, awesome.’ (P10 _NP)

The roles of these important emotions, hope, and empathy were further supported through some of the key stakeholders’ responses. The opportunity to give is presented through empathy. One stakeholder confirmed the following:

‘I think the biggest thing for those patients is the feeling of giving, the feeling of potentially helping those patients who come after them. We were very upfront and talk about our research trials with our patients and the fact that we do not know if the trial will be beneficial to them. Our trials are typically placebo-controlled, so we do not know if they are on placebo or active drugs. We talk about that in-depth with them, but they’re very anxious to do anything they can to help those who come after them.’ (ALS Stakeholder_7)

Similarly,

‘I think people, when they enroll, are actually really hopeful that they’re going to have slowed disease progression. I think it’s mostly positive things that I hear, and some of them, I think too, will be pretty open if you think it is helping. Like if they say, ‘Well, I had my infusion, and then for a few days later, I felt better.’ I think most of the time, it is pretty helpful. I think a lot of people, when I hear them comment, I hear the words, ‘I know this is likely not going to help me, but it will help people down the road.’’ (ALS Stakeholder_2)

Moreover, willingness to participate is also motivated by curiosity and the need to know more about ALS. Most patients shared the ALS community’s needs to gain more knowledge on the disease through clinical trials. Some patients also indicated that, through clinical trials, they foresee (or have experienced) the opportunity to be adequately examined by professionals. Through participation in clinical trials, patients shared that they might benefit from access to new treatments and regular medical attention, which they claim is often absent during regular clinic visits.

‘I just like the idea of having my progress monitored and somebody specific checking in with me. I do not care if it is here or there, wherever. It’s kind of helpful to me to see where I am going.’ (P10_NP)

### 3.3. Living with ALS is difficult: making clinical trials appealing

This theme highlights the major preferences and important considerations that could improve and enable clinical trial participation in patients with ALS. Patients shared their desire for important components, such as compensation (reimbursement), trial accessibility and availability, remote participation and telemedicine opportunities, and clinical trial publicity and awareness. One of the most prominent desires shared by the patients was compensation. Participants perceived this as the benefit of participation and a big tradeoff for time, gas, food, hotels, etc. Although participants confirmed that they were aware that some clinical trials compensated patients, making it mandatory to reduce patients’ expenses or increase the value of compensation was preferred by most participants. For a patient who has participated in a clinical trial, increasing compensation will cover other expenses, such as hotel accommodation, especially for long-distance travel. A patient who had previously participated in a clinical trial stated the following.

‘I mean maybe if it was more, we would spend the night each time.’ (P4_PCT)

Using such compensation for lodging and gas could ameliorate the burden of the travel distance and fatigue. This narrative was supported by most stakeholders as important facilitators of participation in the clinical trials.

‘I think probably just either finding ways to provide or reimburse for transportation. Then, not just transportation for them, but I think a lot of people with ALS their caregivers are still working, and so they’re taking time off to help transport them. So, I think it probably does a lot come back to a financial standpoint. Or just assistance with somewhere to stay if they are coming from far out of town.’ (ALS Stakeholder_4)

Increasing the number of clinical trials and making them more accessible to patients was evident in most of the patients’ discussions. Due to the known complexities of ALS, including the rapid progression of the disease that often leads to sidelining patients at the advanced stages of living with ALS from clinical trials, patients advocate for more clinical trials that accommodate ALS patients at various stages of living with the disease.

‘It’s just I know there are more people who would like to participate, but they’re too far along for some of the criteria. I think that’s why I was able to get into initially like this was because I was not too far along for the symptom’s onset. And I think other than maybe letting people know that it’s out there, I guess, and I do not know how you would do that any better other than everything’s on the Internet.’ (P7_PCT)

When asked about their preferences, another patient who had participated in previous clinical trials also stated:

‘More (trials) available for more patients.’ (P3_PCT)

Additionally, creating relevant awareness of available clinical trials through publicity and marketing has been recommended by patients. They suggested that a thorough explanation of the trial protocol or process would facilitate understanding and improvement of trial enrollment. Others mentioned that sharing stories about past participants’ experiences could also help normalize the ideas of clinical trials. Highlighting clinical trial services that might not otherwise be available outside of such settings would also be beneficial. Creating relevant awareness will increase participation as some patients claimed to have limited information.

‘[…] I did not even know what was available out there, maybe just making it more public, like what’s an option […] I am in an ALS group on Facebook, and a lot of people are asking different questions like, ‘Anyone tried this, or anyone been on this trial or anything like that?’ And so, I have heard the names of different drugs out there that are being tried; I just do not know. How do you get into them unless you have someone who ask you from the clinic? I don’t know. I would not have been able to find it if somebody here had not asked.’ (P10_NP)

Some patients also shared their preferences for remote participation and telemedicine opportunities to make clinical trials more accessible while focusing on patient needs and desires. Although most of the patients understood that the absolute use of telemedicine seems unachievable due to some of the clinical trial procedures, such as blood draw or other laboratory testing, most of the patients were willing to explore such avenues, if possible, or available with clinical trials. Most patients value and envision telemedicine as a prospective improvement in ALS patient care. This idea and preference for remote opportunities stems from patients’ need for reduced stress from clinic visits and distance. Some patients shared that this would encourage participation because of the drastic reduction in physical visits to the clinic, less burden on travel, and reduced fatigue and stress for caregivers. A patient indicated:

‘I don’t want to go anywhere […] I don’t like going into the hospital […] Online’. (P9_NP)

From a similar but slightly different perspective, another patient, while supporting the idea of telemedicine, recounted an unfavorable prior experience with telemedicine. Despite this, the patient acknowledged its potential value and advocated for its inclusion in ALS clinical trials:

‘As it turned out, we did that [*telemedicine*] once. From a standpoint it would have been a remote visit. This might have been repeated except that we did not have a particularly good result from the particular test. Telemedicine is good in general. Now, if they can do the thing with a video call, that would be fine.’ (P3_PCT)

Moreover, contrary to the majority preference for telemedicine, one patient declined such opportunity. This decision was based on the fact that apart from the invariable procedures that necessitate patients’ physical presence, the patient values the physical interaction with other people outside the home and familiar settings, especially to avoid loneliness and for thorough physical examination. Moreover, despite the comfort or convenience that telemedicine could provide for ALS patients, some stakeholders argue that most of the technology in patient homes and some hospitals need upgrading, as outdated machines often contribute to frustration in ALS care. Additionally, telemedicine is dependent on the technological savviness of the patient or caregiver and the technical skills of the staff, which are often absent. If not entirely, one of the stakeholders suggested incorporating telemedicine into clinical trials:

‘Looking at ways to do remote consenting or electronic consenting is a big thing. If these trials can be designed, they can be performed remotely. So, if patients do not want to visit a clinic, telemedicine can be used to digitally perform some of these visits. If there are ways to ship [the] study drug to them, then have them ship back their bottles when the time has arrived. I think that this will help with trials in general. It is just how we can reduce the burden on patients who have to come back physically and what we can do with them remotely or digitally will help. Then, with ALS, too, I think that would also help our ALS trials. However, some of our studies required frequent visits. There’s just no way around it, but if some of them could be done remotely, I think it will really help families as well.’ (ALS Stakeholder_6)

Eventually, for convenience, this may create a positive mental health experience for some patients with ALS and their caregivers.

## 4. Discussion

The diversity and richness of the emerged themes based on the corroboration of the subjective narratives of the patients and key stakeholders significantly contributed to the knowledge of the barriers, preferences, and needs of ALS patients in clinical trials. This study identified and filled a significant gap in ALS patient preferences and decision-making regarding enrollment in clinical trials. By exploring ALS patients’ preferences and decision-making in clinical trials, this study provides specific insights into the value and importance of understanding ALS patients’ unmet needs for meaningful inclusion and consideration. As presented in the findings, the shared emotions have significant impacts on ALS patients’ health-seeking decisions and effective disease management within the clinical setting. Understanding the value of emotions in ALS patients will facilitate the need for emotional and clinical support.

Linked to their shared narratives of frustration, anxiety, fear, and concerns caused by issues such as fatigue, travel distance, and availability of trials, participants emphasized these as major barriers to participation. The findings on these barriers to clinical participation support existing ALS research [13,29,37,38], and studies on barriers to clinical trial participation among cancer patients [26,27,29]. However, these challenges are particularly pronounced for ALS patients due to the disease’s rapid progression and physical demands. Both patients and key stakeholders consistently highlight fatigue as a major concern, as many ALS patients report struggling with excessive fatigue and long travel distances, making the addition of research appointments to their regular medical visits particularly burdensome, which often limit patients’ clinical trial participation [38]. Moreover, fatigue, a known side effect of ALS, not only aggravates disease progression but also intensifies the difficulty of participating in clinical trials, especially when lengthy drives and extended clinic visits are involved [18]. However, these barriers are not isolated as they reflect a broader systemic issue that is rooted in the limited funding of ALS research. Limited financial resources restrict the development of accessible, patient-centered trial designs and infrastructure, especially in rural areas [13].

Another prominent finding of this study indicates the important role of hope and empathy in ALS care. Although this finding contradicts existing arguments on ALS patients’ emotions regarding hope [39,40], it is fascinating to understand ALS patients’ outlook on potential relief or solutions that may be useful for future patients. While considering the differences in research populations, sites, and related bias, which may significantly influence outcomes and generalizability, the analysis and contextual understanding of this finding portray optimism. Patients’ willingness to contribute to ALS discovery and innovation through participation in clinical trials highlights the role of affective and cognitive empathy in health spaces, as it shows the comprehension of the needs of others and willingness to be generous through participation in trials [41]. Paradoxically or ambiguously within the clinical setting, the expression of hope and empathy signifies that despite the complexities of living with ALS and other evident challenges, such as the rapid progression of the disease and its undetermined etiology and cure, the focus on the utilitarian need for improved quality of life remains prominent.

While the analysis and argument towards the role of empathy and hope may be regarded as ‘*pie in the sky*’ or ‘*silver bullet,’* these emotions nonetheless represent ALS patients’ yearnings and desires for positive health outcomes eventually. These desires are further emphasized in patients’ preferences for an increased number of clinical trials, publicity, telemedicine, trial availability, and compensation. These findings on the role of hope and empathy fill an important gap in possibly laying the foundation for patients to feel validated and express their emotions when they would otherwise not. Overall, the findings have provided valuable insights into patients’ willingness and preferences, which will hopefully help healthcare providers, clinicians, and researchers focus on the development of patient-centered care and improve clinical trial participation, resulting in inclusive patient outcomes for people living with ALS.

## 5. Conclusion

Patient enrollment and participation in clinical trials are fundamental to the discovery and advancement of relevant and innovative treatments for ALS. This study was driven by our desire to learn more about our ALS patients’ preferences and attitudes towards clinical trial involvement, particularly considering our rural patient population. To improve participation, this study utilized a patient-centered approach to explore and understand interviewed patients’ experiences and preferences for clinical trial participation. By engaging with these ALS patients’ narratives while incorporating relevant key stakeholders’ feedback, this study provides a novel and insightful analysis of the role of emotions in the decisions and preferences of patients with ALS. In relation to patients’ subjective experiences, the findings of this study identify ALS patients’ challenges, willingness, and preference to participate in clinical trials. Through this analysis, an understanding of the impact of patients’ emotions, such as fear, hope, anxiety, desire, curiosity, and empathy, has presented an avenue that may influence opportunities to address the unmet needs and preferences of ALS patients and implement relevant strategies to address barriers.

To ultimately improve ALS patients’ enrollment and participation in clinical trials, some of the recommended strategies based on the findings of this study include financial reimbursement of patients to mitigate travel barriers and expenses for clinical trials and/or incorporating telemedicine in trials to reduce the need for frequent travel. In addition, the expansion of the number of trial sites, particularly in underserved/rural areas, and the expansion of eligibility criteria can increase patient enrollment and participation, resulting in a more diverse patient population and reduction of unnecessary exclusions. In addition, building on the findings of this study and considering the heterogeneity of ALS, the development of an assessment tool is recommended to better understand the specific clinical trial needs and preferences of ALS patients. This assessment tool would facilitate the identification of tailored strategies to enhance patient enrollment and participation in clinical trials, ultimately contributing to more effective and patient-centered research outcomes. Although not explicitly explored in this study, the role of the caregivers cannot be overemphasized. To better provide the needed support and health outcome for ALS patients, the needs and preferences of their caregivers could also be explored through the assessment tool and related research. In addition, to address most of the shared barriers meaningfully, especially the root cause of limited funding, there is a critical need for targeted and sustained funding that supports clinical trials, transportation assistance, and possibly home-based participation options. Investing in these areas would not only reduce enrollment and participation difficulties but also improve engagement in ALS clinical research.

It is imperative to highlight some limitations of this study. The findings of this study are not representative of the broader ALS patient and stakeholder population as the small sample size limits generalizability. Nonetheless, engaging the high information power of the small but qualitative sample size contributes to the relevant data on this population. Additionally, the study was conducted at a specific site in the United States, which may not capture the diversity of experiences and healthcare practices in different regions and locations, thus affecting the applicability of the findings to other settings. Despite the team’s rigorous, systematic analysis and reflective efforts during data analysis and writing, the influence of subjectivity inherent in qualitative research is acknowledged in this study. This study captures a specific period in time and may not reflect changes in patients’ conditions or stakeholders’ perspectives over time, suggesting that additional and longitudinal studies could provide more insights into the evolving nature of ALS and its impact on patients’ decision-making. Despite these limitations, this study provides substantial insights from diverse but relevant perspectives, offering valuable guidance that could facilitate improvements in ALS clinical trial development and enrollment.

## Supplementary Material

Supplementary Material.docx

## Data Availability

The data that support the findings of this study are available upon reasonable request. The data are not public due to ethics restrictions.
