# Supplementary material for: ‘I want to be generous, but I only have limited energy’: a qualitative study of amyotrophic lateral sclerosis patients’ preferences for clinical trials participation
Source: Ann Med. 2025 Nov 11;57(1):2586150. doi: 10.1080/07853890.2025.2586150 (PMC12608098; doi:10.1080/07853890.2025.2586150)
Supplement: Supplementary Material.docx [file IANN_A_2586150_SM3915.docx]

**Themes, subthemes and related quotations**

| Themes | Subthemes | Quotations |
| --- | --- | --- |
| The disease itself: Barriers to clinical trial participation | Frustration: Fatigue and travel distance | “I want to be generous, but I only have limited energy.” (P9_NP).  “The travel is hard […] I mean it's a long drive, and it's a miserable drive. It's not easy […] You need a lift to get in and out bed […] Nothing is easy for us. Like everything is complicated, traveling, trying to figure out what to do about me going to the bathroom, sleeping arrangement, all that kind of stuff” (P10­_NP) |
|  | Anxiety: Availability of trials | “I just feel like we're spinning our wheels until something is potentially approved that may be considered a cure. And I know every ALS patient I'm sure has said this or family or caregiver, it's just frustrating to say the least”. (P6_NP)  “I guess just the trial availability was one of the factors in not participating”. (P10_NP) |
|  | Fear: Eligibility requirements   - - Disease progression | “I hate that I had to wait this long, honestly […] It's been seven months that I've waited to get in and that's a long time. I've gone from being able--I was walking the first time they asked me about the ALS trial with like a little weakness in my legs, but now I'm like, I'm done with everything but my arm. So, it's like I've lost a lot in the seven months. I wish I would've gotten in back then. But it is what it is.” (P10_NP)  “I submitted a request, a trial. It's not really a request, but it was a trial like requirements. There was like six things I needed to answer. And when we got to my dad, everything was fine for like the first five, but the sixth one is when they, wanted to do a, I can't remember the official name for it, but it was like a breathing test to see if he could ... capacity test…50% or above. Dad is on a trach, I know there's no way he could pass that. So, I've never heard a response other than from XXX from the XXX ALS that, well, maybe we need to look at some different options…” (P6_NP) |
|  | Fear: Trial effectiveness | “It was just kind of disheartening knowing it didn't do anything.” (P7_PCT)  “The study is unlikely to benefit me”. (P9_NP) |
|  |  | **Stakeholders** |
|  |  | “Unfortunately for this group there are several barriers. They are very anxious about participating in the trials. However, many of our patients do not live locally. Most of them do not, so transportation to and from hospitals can be difficult. As patients progress to ALS, they become fatigued more easily, so visits for research trials can be lengthy […] Some patients right off the bat want to be included in research trials as soon as they are diagnosed. However, more often than not, patients just need to wrap their heads around it and get their lives in order. Once they start progressing, they think about the research trials. The difficulty with this is that many trials have these criteria that the patients have to meet. The major one is breathing. They must be above a certain percentage in this breathing assessment. This has been a challenge because patients sometimes progress rapidly, and by the time they are coming into a research trial, they may not qualify.” (ALS Stakeholder_7)  “I think geography is a huge one. A lot of our patients are driving a couple hours to get there. That can be a pretty taxing thing for anybody, let alone if you have ALS, to drive to clinic for a couple hours. Then typically, patients are seen in clinic by multiple providers, so they might be there for two to three hours and then have to drive home. So, I think that can be a limiting factor”. (ALS Stakeholder_1) |
| The bigger picture: Willingness to participate in clinical trials. | Empathy: The role in healthcare decisions | “My main consideration was, even if it didn't help me, it might help someone in the future, so that was always on my mind… I wanted to be helpful and hopefully get help myself.” (P4_PCT) |
|  | A wave of hope: Finding solutions | “In hopes that maybe there was something that would help slow the progression and if not, at least help gather information that maybe down the road that would help, if not me, somebody else. I mean, we knew it wasn't going to be a cure, so it was just to help somebody else. That's what we talked about. We knew it wasn't going to--, yeah, it wasn't the silver bullet, so to speak, but we knew maybe there was a chance.” (P7_PCT) |
|  | Curiosity: Gain knowledge on ALS | “Well, I know that it's probably not likely that it's necessarily going to help me specifically, but even if this whatever I try is something that doesn't work, it's just one more thing that we know doesn't work. Trying to get that much closer to answers, doing whatever I can to help with that [...] It’s one of those--, like I want to participate because I want to be helpful in whatever way that I can. And if it helps me, awesome.” (P10_NP) |
|  | Motivation: Detailed examination | “I just like the idea of having my progress monitored and somebody specific checking in with me. I don't care if it's here or there, wherever. It's kind of helpful to me to see where I'm going.” (P10_NP) |
|  |  | **Stakeholders** |
|  |  | “I think the biggest thing for those patients is the feeling of giving, the feeling of potentially helping those patients who come after them. We're very upfront and talk about our research trials with our patients and the fact that we don't know of the trial if it will be beneficial to them. Our trials are typically placebo controlled, so we don't know if they're on placebo or active drug. We talk about that in depth with them, but they're very anxious to do anything they can to help those who come after them.” (ALS Stakeholder_7)  “I think people, when they enrol, are actually really hopeful that they're going to have slowed disease progression. I think it's mostly positive things that I hear, and some of them, I think too, will be pretty open if you think it's helping. Like if they say, ‘Well, I had my infusion and then for a few days later I felt better.’ I think most of the time it's pretty helpful. And I think a lot of people, when I hear them comment, I hear the words, ‘I know this is likely not going to help me, but it'll help people down the road.’ But I think people are very cognizant of that and realize the importance of it.” (ALS Stakeholder_2)  “It definitely helps to improve what options we have for treatment because we have been part of clinical trials, there's another medicine that can be offered to patients now that we didn't have 10 years ago. It's helping to continue to improve what are some medications can we use to maybe help slow down the progression of ALS. We don't have a cure for ALS at this time, so clinical trials do give all of us hope. What can we do to improve the care of our patients and are there new medications or treatments out there that we can use to help slow down the progression of ALS. We were recently part of a clinical trial that was positive and so that's great that through these research studies we can continue to help move forward the science and hopefully help other ALS patients in the future based off the current ones who participate in the study”. (ALS Stakeholder_6) |
| Living with ALS is difficult: Making clinical trials appealing. | Desire: Compensation | “I'm thinking more of like some sort of gesture of thank you for doing this [participation]... Like, “Here thanks for your—,” because I'm going to have an infusion in my arm once a week. It's not exactly going to be easy or convenient or comfortable for me […] and I doubt that I personally see a benefit for it. So, it would be nice to have a thank you for it […] Yeah. Compensation would be nice […] Gas cards, like whatever, I don't even care, like some sort of something.” (P10_NP)  I think probably just either finding ways to provide or reimburse for transportation. And then not just transportation for them, but I think a lot of people with ALS their caregiver is still working, and so they're taking time off to help transport them. So, I think it probably does a lot come back to a financial standpoint. Or just assistance with somewhere to stay if they're coming from far out of town. (ALS Stakeholder_4) |
|  | Desire: Increase number of trials   - Close proximity and accessibility | “I know there are more people who would like to participate, but they're too far along for some of the criteria. And I think that's why I was able to get in initially, this was because I wasn't too far along for the symptom’s onset. And I think other than maybe letting people know that it's out there, I guess, and I don't know how you would do that any better other than everything's on the internet.” (P7_PCT)  “More (trials) available for more patients.” (P3_PCT)  “I didn't even know what was available out there or making it more public, like what's an option. I know a lot of, I'm in an ALS group on Facebook, and a lot of people are asking different questions like, ‘Anyone tried this or anyone been on this trial or anything like that?’ I've heard the names of different drugs out there that are being tried, I just don't know. How do you get into them unless you have somebody who asks you from the clinic? I don't know. I wouldn't have been able to find it if somebody here hadn't asked.” (P10_NP)  “I don't want to go anywhere […] I don't like going into the hospital […] Online”. (P9_NP)  “As it turned out, we did that [*telemedicine*] once. From a standpoint it would have been a remote visit. This might have been repeated except that we did not have a particularly good result from the particular test. Telemedicine is good in general. Now, if they can do the thing with a video call, that would be fine.” (P3_PCT) |
|  | - - Create awareness and publicize trial availability. |  |
|  | - - Telemedicine |  |
|  |  | **Stakeholders** |
|  |  | “I think probably just either finding ways to provide or reimburse for transportation. And then not just transportation for them, but I think a lot of people with ALS their caregiver is still working, and so they're taking time off to help transport them. So, I think it probably does a lot come back to a financial standpoint. Or just assistance with somewhere to stay if they're coming from far out of town”. (ALS Stakeholder_4)  “So, I would say coordinating appointments with already scheduled clinical visits so that the travel…less burden on travel. Developing telemedicine outcome measures so that patients can participate from their home”. (ALS Stakeholder_3).  Looking at ways to do remote consenting or electronic consenting is a big thing. If there's ways that these trials can be designed, so things can be done remotely. So, if patients don't want to come in for a clinic visit if we can use telemedicine to do some of these visits with them digitally. If there's ways that we can ship steady drug to them and then have them ship back their bottles when the time has arrived. I think that will help trials in general. It's just how can we reduce the burden on patients of having to physically come back and what can we do with them remotely or digitally will help. Then with ALS too, I think that would also help our ALS trials. Some of our studies, it unfortunately requires frequent visits. There's just no way around it, but if some of them could be done remotely, I think it will really help families as well (ALS Stakeholder_6)  And so, I think flyers and printed out things might be a little bit better option. And addressing them directly instead of sending one thing right to, "an entire clinic." Like, "Oh, this is going to go out to internal medicine to hang up." Maybe having it be directly to the providers and addressed to them. I think I would be a little bit more apt to pay attention then. (ALS Stakeholder_2) |

**P1-10 – Patient 1 to 10

**NP – No clinical trial participation

**PCT – Participated in clinical trials
